# Total saponins from the leaves of *Panax notoginseng* inhibit depression on mouse chronic unpredictable mild stress model by regulating circRNA expression

**DOI:** 10.1002/brb3.1127

**Published:** 2018-10-09

**Authors:** Hualin Zhang, Ziming Chen, Zhiyong Zhong, Weifan Gong, Jun Li

**Affiliations:** ^1^ School of Chemistry and Chemical Engineering Lingnan Normal University Zhanjiang China; ^2^ Guangdong Medical Laboratory Animal Center Guangzhou China; ^3^ School of Pharmaceutical Sciences South‐central University for Nationalities Wuhan China

**Keywords:** chronic unpredictable mild stress, circRNAs, Depression, hippocampus, *Panax notoginseng*, ventral medial prefrontal cortex

## Abstract

**Objectives:**

Total saponins from the leaves of *Panax notoginseng* saponins (SLPN) could inhibit development of depression, but the underlying mechanisms remains unclear. This study aimed to address the roles of circular RNAs in depression inhibition by SLPN.

**Methods:**

The mouse chronic unpredictable mild stress (CUMS) model was established, which were confirmed by mouse weight, forced swimming test (FST) and tail suspension test (TST). Effects of SLPN on depression were evaluated in CUMS through these same assays. Circular RNA profiles in mouse ventral medial prefrontal cortex (VMPC) and hippocampus of CUMS mice were determined by high‐through sequencing, followed by confirmation via qRT‐PCR. Overexpression of mmu_circ_0001223 was done by transfection of PC12 cell through lentiviral system. Protein abundances of cAMP response element binding protein 1(CREB1) and brain‐derived neurotrophic factor (BDNF) were evaluated by western blotting.

**Results:**

Mouse body weight, immobility time in FST and immobility time in TST of CUMS mice were significantly recovered by SLPN treatment. A large number of circular RNAs were differentially expressed in the ventral medial prefrontal cortex (VMPC) and hippocampus tissues of CUMS mice. Among them, mmu_circ_0001223 expression was greatly decreased in CUMS mice, but significantly elevated by SLPN treatment. The protein levels of CREB1 and BDNF were also remarkably promoted in CUMS mice by treatment of SLPN. Overexpression of mmu_circ_0001223 enhanced CREB1 and BDNF protein levels in PC12 cells.

**Conclusion:**

SLPN regulate the expression of large number circular RNAs in CUMS mice, which might be important mediators of SLPN's anti‐depression effects.

## 1 INTRODUCTION

Depression is a disorder of low mood and aversion to activity, which could severely affect persons’ thoughts, feelings and behavior, even develop into death by suicide (Jukić and Arbanas, 2013). The depressed mood could be caused by life events like loss of relatives, side effect of certain medications, or symptom of other severe physical diseases such as diabetes, hypoandrogenism, hypothyroidism and even cancers (Li, Fitzgerald, & Rodin, 2012; Rustad, Musselman, & Nemeroff, 2011; Saravane et al., 2009). Patients with depression could present with extremely sad and anxious mood, hopeless and worthless feelings, and other symptoms like senses of guilt, anger, irritability, ashamedness and restlessness, which is expected to be the second major cause of disability in coming years (Mohit, 2006). Recently, depression management in clinics largely depends on psychotherapy and medications including selective serotonin re‐uptake inhibitors (SSRI) such as fluoxetine and sertraline, and 5‐serotonin and norepinephrine reuptake inhibitors (SNRI) like venlafaxine and duloxetine (Fava, 2002). However, it has been suggested that antidepressants should not be routinely administrated for mild depression due to side effects and poor risk‐benefit ratio, and also for children and adolescent patients because of the uncertainty around their therapeutic advantages (Cipriani et al., 2016). To meet the increase of depression incidence worldwide, development of novel anti‐depression medications with high efficacy and low side effects is an urgent need for the research community.

Natural active molecules from medicinal plants have been currently shown as promising sources of new anti‐depressants. For instance, previous reports demonstrated the potential depression‐ameliorating effects of many herbs and plant‐derived chemicals such as Butea superba, Radix Polygalae, Curcumin and natural polyphenols that have long been applied in traditional medicine (Liu et al., 2010; Mizuki et al., 2014; Pathak, Agrawal, & Dhir, 2013; Seo et al., 2015). Among them, total saponins extracted from caudexes and leaves of a traditional Chinese medicinal plant *Panax notoginseng* exhibited effective inhibitory function against depression in animal models, which might be mediated by regulation of brain monoamine neurotransmitters and Ca2^+^ concentration (Dang et al., 2009; Xiang et al., 2011). Subsequent studies revealed that many components of *P. notoginseng* saponins act as the major bioactive players of the anti‐depression effects of *P. notoginseng* extracts such as ginsenosides Rb3, Rb1 and Rg1, partially mediated by brain‐derived neurotrophic factor (BDNF) pathway (Cui, Jiang, & Xiang, 2012; Jiang et al., 2012). Moreover, our previous investigations showed that deglycosylated derivative of ginsenoside Rb3 (Rg3) performed its depression‐inhibiting roles by modulating signaling pathways associated with BDNF and cAMP response element binding protein (CREB) using cellular and animal models (Zhang et al., 2016; Zhang, Zhou, Chen, Zhong, & Zhong, 2017). In consideration of the complex constitution of *P. notoginseng* extracts and the complicated pathological processes linked with depression, the molecular mechanism mediating anti‐depression effects of total saponins from the leaves of *P. notoginseng* (SLPN) remains far from being fully understood.

Circular RNAs (circRNAs) are newly identified noncoding RNA molecules that regulate gene expression as miRNA sponges or associating with RNA binding protein (RBP), thus being involved in pleiotropic physiological and pathological functions (Hanan, Soreq, & Kadener, 2016; Le et al., 2015). Over thousands of circRNAs were detected in various organ and tissues in multiple species, and large number of circRNAs were found to be expressed in brain tissues and neurons, suggesting their critical roles in neuron function maintenance and pathological processes of brain‐related disorders such as neurodegenerative diseases (Hanan et al., 2016). For instance, circRNA Cdr1as was binding with two miRNAs in human and mouse brain tissues, and the loss of circRNA Cdr1as could results into degradation of specific miRNA targets and dysfunctional synaptic transmission, which is associated with normal brain function and neuropsychiatric disorders (Piwecka et al., 2017). More importantly, recent analysis of patients with type 2 diabetes mellitus (T2DM) and depression showed that over 200 circRNAs were significantly differentially expressed in T2DM group complicated with depression, showing the possible involvement of circRNAs in both depression and T2DM pathologies, as well as the depression associated with T2DM (Jiang et al., 2017). However, the specific roles of individual circRNAs in depression pathogenesis and the underlying molecular mechanisms are still largely unknown. Also, whether circRNAs are involved in the inhibition of depression by SLPN and other bioactive components have not being addressed before.

In the study, we established the chronic unpredictable mild stress mouse model to evaluate the roles of circRNAs in depression inhibition. Differentially expressed circRNAs in mouse ventral medial prefrontal cortex (VMPC) and hippocampus tissues induced by SLPN were detected by deep sequencing, followed by expressional and functional validations, which would provide novel insights into the implication of circRNAs in depression treatment with bioactive saponins.

## 2 MATERIAL AND METHODS

### 2.1 Extraction of Total saponins from the leaves of Panax notoginseng saponins (SLPN)

**Figure 1:**
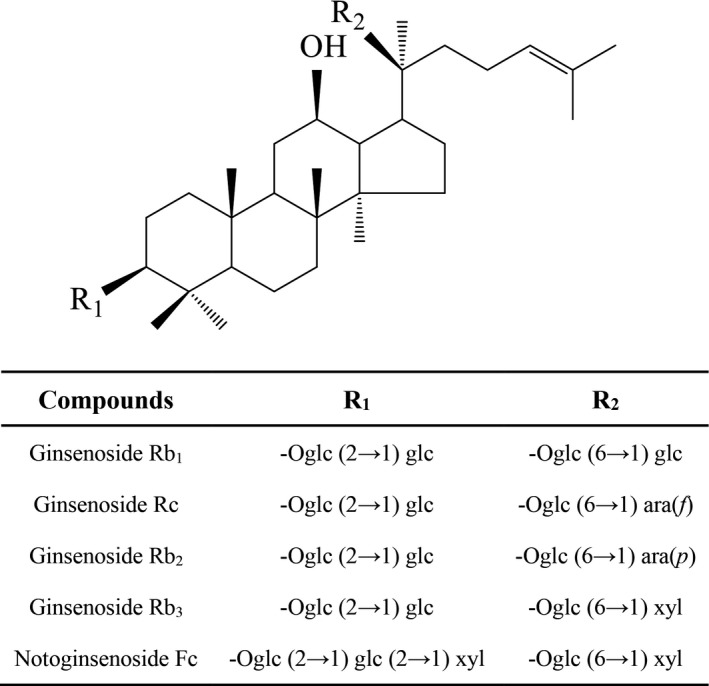
Chemical structure of ginsenosides Rb1, Rc, Rb2, Rb3 and notoginsenoside Fc

Total saponins from the leaves of Panax notoginseng saponins (SLPN) were extracted and purified in our lab, meeting the criterion of the Pharmacopoeia of the People's Republic of China, 2015. Saponins in SLPN including ginsenoside Rg1, Rb1, Rc, Rb2, Rb3, Rd, F1, Rg3, compound K, Rh2, 20(S)‐protopanaxadiol, and notoginsenoside R1, Fa, Fc, Fe, and so on, were identified by high performance liquid chromatography (HPLC). The contents of the major components Rb1, Rc, Fc, Rb2, and Rb3 were 2.37%, 9.78%, 7.94%, 2.85%, and 15.73%, respectively, and their chemical structure were shown in Figure 1. Additionally, the total flavonoid content was 0.34% determined by ultraviolet spectrophotometry. Alkaloids, polysaccharides, starches, tannins, proteins, amino acids, and peptides were not detected by the typical colorimetric method.

### 2.2 Animal and cell culture

Twenty‐six C57BL male mice used for model establishment were purchased from the Experimental Animal Center of Sun Yat‐Sen University (Guangzhou, China). PC12 cells (ATCC; USA) were cultured in RPMI1640 medium supplemented with horse serum and fetal bovine serum (FBS) at 37°C in a humidified incubator supplied with 5% CO_2_.

### 2.3 Animal model and treatment

Twenty‐six C57BL male mice aging 6–8 weeks were used for the establishment of chronic unpredictable mild stress (CUMS) mouse model, and they were randomly divided in to three groups, CUMS groups (10 mice), CUMS+SLPN group (10 mice) and NC group (six mice). The whole procedures of establishment of the chronic mild stress (CMS) model were carried out as previously introduced (Zhang et al., 2017). Briefly, each mouse was randomly given one of the following unpredictable mild stressors per day: wet bedding for 24 hr, behavior restriction for 2 min, water deprivation for 24 hr, food deprivation for 24 hr, tail nip for 5 min or cage tilting for 24 hr, lasting for totally four weeks. Finally, mouse weight, forced swimming test (FST) and tail suspension test (TST) were used for evaluation of the animal models. For analysis the effects of SLPN, the CUMS mice were randomly grouped into the CUMS groups and CUMS+SLPN group, which were intragastrically administrated one dose of 30 mg/kg saline or SLPN respectively each day, lasting for 3 weeks. Another six male mice aging 6–8 weeks were applied as the negative control group (NC group). All animal studies have been approved by The Institute Research Medical Ethics Committee of Sun Yat‐Sen University. Animal handling procedures in this study were done strictly following the Guidelines for Laboratory Animal Care and Use of Sun Yat‐Sen University.

### 2.4 Behavior tests

Forced swimming test (FST) and tail suspension test (TST) in this study were carried out as previously introduced (Zhang et al., 2017). For FST assay, mice were transferred to glass cylinders filled with 10 cm water 2 hr after corresponding intragastric administrations, kept at 24 ± 1°C for 6 min and immobility duration of mice were detected during the final 4 min. Immobility time were defined by observation an obvious behavior of floating motionless or making only those movements necessary to keep their head out of the water. For TST analysis, each mouse was separately suspended in a cage (25 cm × 25 cm × 30 cm) by its tail using a 1 cm‐long adhesive tape 2 hr after intragastric administration. Mouse heads were kept 15 cm away from the cage bottom. Analysis was carried out in a dark room to minimize noise interference, with mice separated from each other to avoid visual or acoustic communication between mice. Behaviors of mice were recorded using video cameras, and immobility was defined by no movement during the whole test lasting for 5 min.

### 2.5 Circular RNA profiles by deep sequencing

Expressional files of circular RNAs in mouse ventral medial prefrontal cortex and hippocampus tissues, as well as their relative levels, were determined by next‐generation sequencing as previously described (Ye et al., 2017). After corresponding treatment, mice were sacrificed for collection of cortex and hippocampus tissues through the standard protocols. Collected mouse tissues were immediately homogenized with liquid nitrogen, and total RNA samples from each samples were extracted using the Animal Tissue RNA Purification Kit (#25700, Norgen Biotek Corporation) as instructed by the manufacturer. After removing rRNA and linear RNA ingredients using rRNA‐binding magnetic beads and RNase R (#RNR07250, Epicentre) respectively, the remaining RNAs were then subjected to synthesis of cDNA with random primers, which were analyzed using Agilent 2100 Bioanalyzer System and Illumina sequencer for sequencing and quantitation. Sequencing raw data that went through quality control, and clean reading after filtering were blasted against the circBase reference sequences by experienced bioinformatists. Finally, numbers of clean reads matching reference sequences were recorded, and expressional levels and differentially expressed circRNAs were determined.

### 2.6 Quantitative RT‐PCR

Relative expressional levels of cirRNAs and mRNAs in this study were quantified by quantitative reverse‐transcription polymerase chain reaction (qRT‐PCR) using specific primers. After treatment with corresponding drugs, mouse ventral medial prefrontal cortex and hippocampus tissues were homogenized in liquid nitrogen and total RNA samples were extracted as introduced above. Approximately 2 mg RNA of each sample were then applied to cDNA synthesis using the High‐Capacity cDNA Reverse Transcription Kit (Cat#: 4368813; Thermo Fisher Scientific, CA, USA) following the manufacturer's instructions. The relative expression of RNA molecules were finally determined by PCR using the SYBR Select Master Mix kit (Applied Biosystems, Cat: 4472908) as instructed by the manufacturer. Particularly, divergent primers and opposite‐directed pairs of primers were used for measurement of circular RNAs and linear mRNAs respectively. GAPDH gene was applied as the internal standard for relative quantitation. At least three biological and technical replicates were carried out the statistical analysis of RNA levels.

### 2.7 Cell transfection

Expression of circRNA in PC12 cells was finished as previously described (Li et al., 2015). The circ_0001223 cDNA synthesized by the Nuclee Company (Guangzhou, Guangdong, China) were ligated with pLVX‐IRESneo lentiviral expression vector (Clontech Laboratories Inc., San Francisco, USA). The sequence of cDNA in lentiviral expression vector was confirmed by direct sequencing, and then transfect PC12 cells as previously described (Li et al., 2015). Expression of circ_0001223 in PC12 cells were checked by qRT‐PCR and western blotting.

### 2.8 Western blotting

PC12 cell lines were collected by centrifuge at 800 g for 5 min and washed three times with PBS, mixed with protein sample loading buffer and boiled 5 min at 100°C. Total proteins were then separated by 10% SDS‐PAGE and electro‐blotted onto PVDF membranes, blocked with 5% lipid‐free milk solution for 2 hr, incubated with primary antibodies diluted in TBST for 1 hr at RT, washed three times with PBS for 10 min, incubated with secondary antibodies diluted with TBST, and finally development with ECL solution (Amersham™) for quantitation of protein relative abundances. At least three biological repeats were done for relative expression. GAPDH was applied as the internal standard in western blotting assay. The following antibodies purchased from Abcam (UK) were used in this study: Anti‐CREB1 antibody (Cat. #ab47781), Anti‐BNDF antibody (Cat. #ab10505) and Anti‐GAPDH antibody (Cat. #ab8245).

### 2.9 Statistical analysis

Data were presented as mean ± SEM. SPSS 18.0 software package was used for the statistical analysis in this study. Student *t*‐test and analysis of variance (ANOVA) were performed for comparison between two groups and over two groups respectively. Significant differences were defined by a *p* value of <0.05.

## 3 RESULTS

### 3.1 Establishment of Chronic unpredictable mild stress model and the reverse effect by SLPN

**Figure 2:**
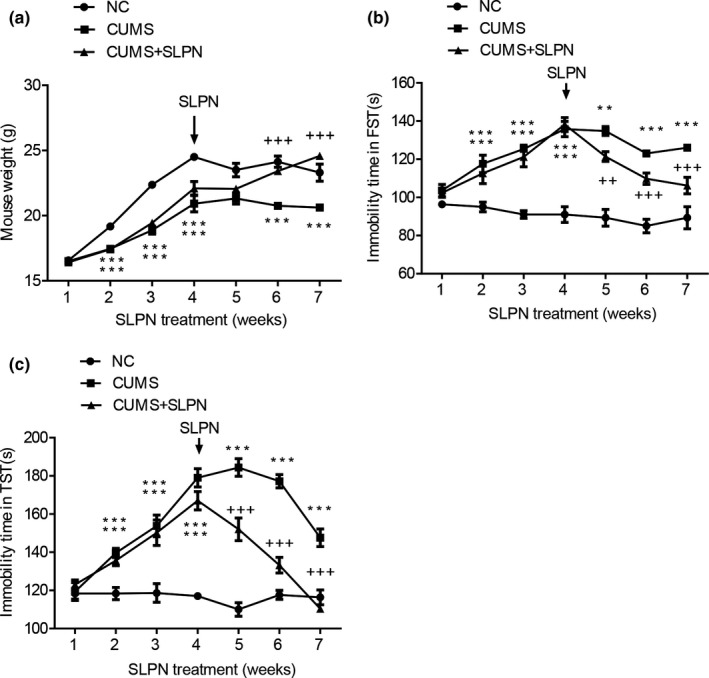
Establishment of mouse chronic unpredictable mild stress model and inhibition of depression by SLPN. (a) Body weights variation in CUMS mice and the reverse effect of SLPN. (b) Immobility time in FST of CUMS mice and the reverse effect of SLPN. (c) Immobility time in TST of CUMS mice and the reverse effect of SLPN. CUMS: chronic unpredictable mild stresse; FST: forced swimming test; SLPN: Total saponins from the leaves of *Panax notoginseng*; NC: negative control; TST: tail suspension test; ** and ***, v.s. NC, refer to a *p* value of <0.01 and <0.001 respectively. ^++^ and ^+++^, v.s. CUMS, refer to a *p* value of <0.01 and <0.001 respectively

For analysis of the effects of total saponins of the leaves of *Panax notoginseng* (SLPN) on depression, mouse chronic unpredictable mild stress (CUMS) model were established as described in the Material and Methods session. The development of depression in treated mice were then evaluated by analysis of mouse weight, immobility time in forced swimming test (FST) and immobility in tail suspension test (TST). From weight curves, we could see that weights of CUMS mice were significantly lowered compared with the negative control group (Figure 2a). Also, the immobility time of CUMS mice was significantly increased in FST assay (Figure 2b). And the immobility time was also significantly increased in CUMS mice during TST assay (Figure 2c). The changes of mouse weight and immobility are typical features of depression, showing the successful establishment of depression model. The CUMS were then applied for the following analysis of the molecular mechanisms underlying anti‐depression roles of SLPN.

Mice in the CUMS model group were treated with SLPN for consecutive 3 weeks, and the behavior properties were analyzed as shown above. We observed that weights of mice treated with chronic unexpected stresses were obviously increased compared with the CUMS group, showing the significant remission of depression symptoms (Figure 2a). In consistence, the immobility time of CUMS mice treated with SLPN were markedly decreased compared with the CUMS group of mice in FST assay (Figure 2b). The significant decrease of immobility was also observed in CUMS mice during the TST assay, in comparison with the CUMS group (Figure 2c). These results showed that treatment with SLPN significantly inhibited the development of depression in the CUMS mouse model.

### 3.2 SLPN alters circRNA profiles in mouse VMPC and hippocampus tissues

**Figure 3:**
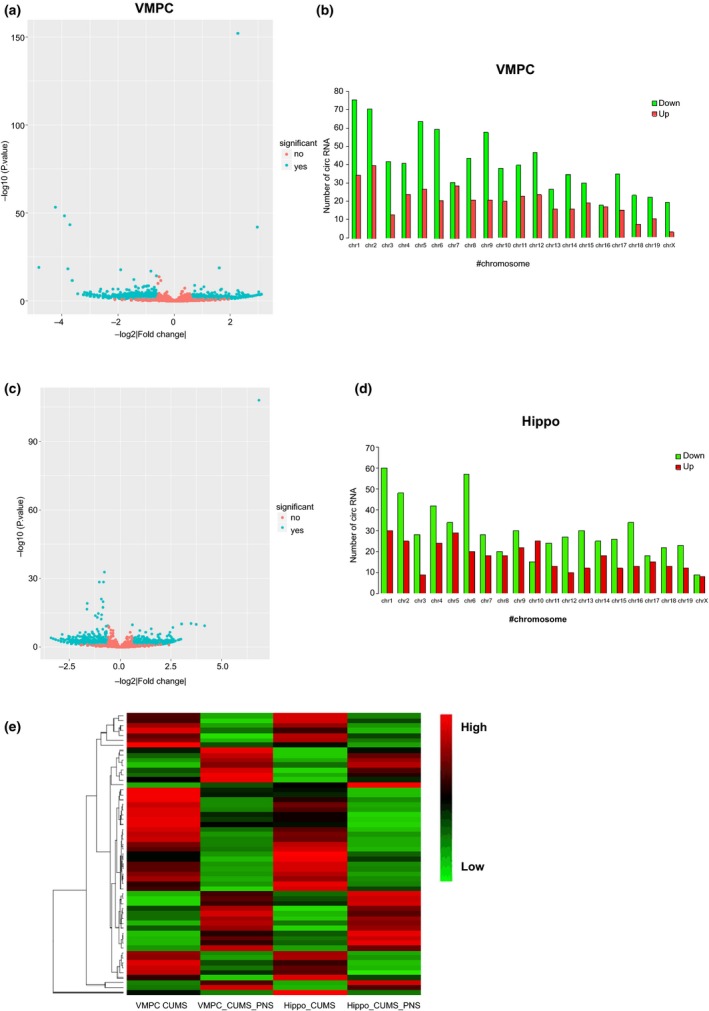
Differential expression of circRNAs in CUMS mice by SLPN treatment. (a) Volcano plot of circRNAs expression in CUMS mouse VMPC tissue. X‐asis: Log2 ratio of circRNA expression levels. Y‐asis: the FDR value (−log10 transformed) of circRNA. (b) Number of differentially expressed circRNAs in VMPC tissues among different chromosomes. Green and red columns represent numbers of down‐regulated and up‐regulated circRNAs respectively. (c) Volcano plot of circRNAs expression in hippocampus tissue of CUMS mice. (d) Number of differentially expressed circRNAs in hippocampus tissues among different chromosomes. (e) Heat map showing the differentially expressed circRNAs in mouse VMPC and hippocampus tissues. VMPC: ventral medial prefrontal cortex; CUMS: chronic unpredictable mild stresse; SLPN: total saponins from the leaves of *Panax notoginseng*

To investigate the molecular mechanisms underlying the depression‐inhibiting roles of SLPN, circRNA expression files in mouse ventral medial prefrontal cortex (VMPC) and hippocampus tissues were analyzed by high‐through sequencing as introduced above. As shown in Figure 3, a large group of circRNAs were differentially expressed in the mouse ventral medial prefrontal cortex of CUMS mice by SLPN (Figure 3a,b). Also, the differentially expressed circRNAs in the hippocampus tissues of CUMS mice treated with SLPN were also in large quantity, compared with the CUMS group (Figure 3c,d). Of note, SLPN could cause both increase and decrease of specific circRNA molecules in mouse brain tissues, showing that the complex regulating networks mediated by various circRNAs (Figure 3e). Furthermore, the majority of circRNAs displayed similar patterns in both the VMPC and hippocampus tissues, suggesting that similar functions might be played by specific circRNAs in these two different tissues during suppression of depression with SLPN (Figure 3e). The large number of differentially expressed circRNAs accompanying depression remission by SLPN suggested that circRNA might performed critical roles in development of depression and anti‐depression effects of SLPN.

### 3.3 Regulation of circ_0001223 by SLPN in CUMS mice

**Figure 4:**
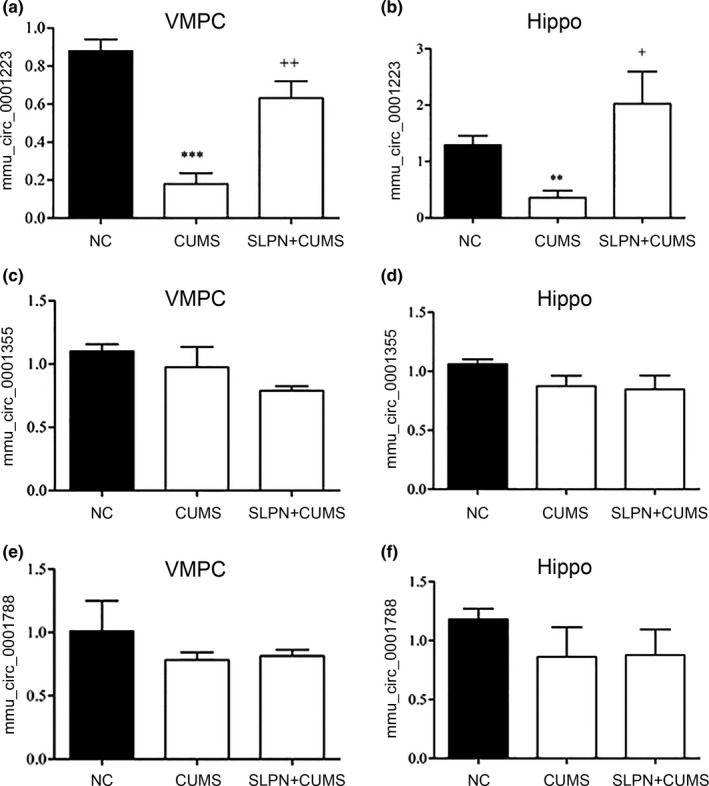
Regulation of mmu_circ_0001223 expression by SLPN. (a, b) Mmu_circ_0001223 expression in VMPC (a) and hippocampus tissues (b) of CUMS mice treated with SLPN. (c, d) Mmu_circ_0001355 expression in VMPC (c) and hippocampus tissues (d) of CUMS mice treated with SLPN. (e, f) Mmu_circ_0001788 expression in VMPC (e) and hippocampus tissues (f) of CUMS mice treated with SLPN. Quantitative RT‐PCR was performed to quantify circRNA levels. CUMS: chronic unpredictable mild stress; NC: negative control; SLPN: total saponins from the leaves of *Panax notoginseng*; VMPC: ventral medial prefrontal cortex; **and***, v.s. NC, refer to a *p* value of <0.01 and <0.001, ^+^and^++^, v.s. CUMS, refer to a *p* value of <0.05 and <0.01

For more insights into the roles of circRNA molecules in inhibition of depression by SLPN, expression of three circRNAs mmu_circ_0001223, 0001355 and 0001788 were further analyzed by quantitative RT‐PCR (Figure 4). We found that the expressional levels of mmu_circ_0001355 and 0001788 were slightly down‐regulated in CUMS mice, but SLPN had no significant effect on their expression levels in both ventral medial prefrontal cortex and hippocampus tissues of CUMS mice (Figure 4c‐f). However, we observed that the expression of mmu_circ_0001223 were greatly suppressed in ventral medial prefrontal cortex of CUMS mice compared with negative controls, while treatment with SLPN significantly recovered the mmu_circ_0001223 level, almost comparable to that in the negative controls (Figure 4a). Meanwhile, mmu_circ_0001223 expression level in mouse hippocampus tissues exhibited similar alteration in CUMS models after being treated with SLPN (Figure 4b). The alterations in mmu_circ_0001223 abundance of CUMS models suggested that this circRNA might be an important regulator of depression, as well as mediator of the depression‐inhibiting functions of SLPN.

### 3.4 CREB1 and BNDF expression regulated by SLPN

**Figure 5:**
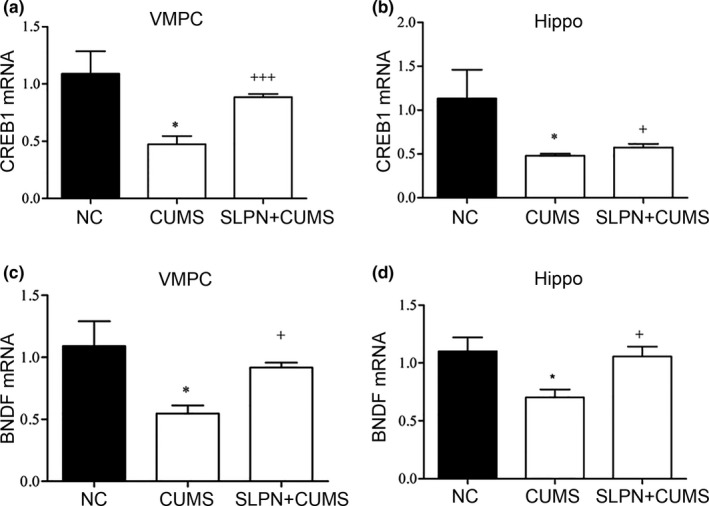
SLPN promotes CREB1 and BNDF expression in CUMS mice. (a, b) CREB1 expression in VMPC (a) and hippocampus tissues (b) of CUMS mice treated with SLPN. (c, d) BNDF expression in VMPC (c) and hippocampus tissues (d) of CUMS mice treated with SLPN. Quantitative RT‐PCR was carried out to quantify gene expression. BDNF: brain‐derived neurotrophic factor; CREB1: cAMP response element binding protein; CUMS: chronic unpredictable mild stress; NC: negative control; SLPN: total saponins from the leaves of *Panax notoginseng*; VMPC: ventral medial prefrontal cortex; *, v.s. NC, refer to a *p* value of <0.05, ^+^and^+++^, v.s. CUMS, refer to a *p* value of <0.05 and <0.001

Previous reports showed the CREB1 and BNDF‐related signaling pathways were involved in the pathogenesis of depression and also depression treatment (Jiang et al., 2012; Zhang et al., 2017). For further validation of anti‐depression of SLPN, the expression of CREB1 and BNDF expression in CUMS mice treated with SLPN were quantitatively analyzed by quantitative RT‐PCR (Figure 4). We observed that relative CREB1 mRNA levels was remarkably depressed in VMPC of CUMS mice, but SLPN treatment effectively elevated CREB1 mRNA level (Figure 5a). The mRNA levels of CREB1 gene in mouse hippocampus tissues showed similar changes induced by both chronic stresses and SLPN treatment (Figure 5b). Similarly, the mRNA levels of BNDF also showed drastic decrease in CUMS model, but greatly up‐regulated by SLPN in both VMPC and hippocampus tissues (Figure 5c,d). The significant changes of these two signaling molecules associated with depression further supported the anti‐depression role of SLPN.

### 3.5 Circ_0001223 promotes CREB1 and BNDF expression

**Figure 6:**
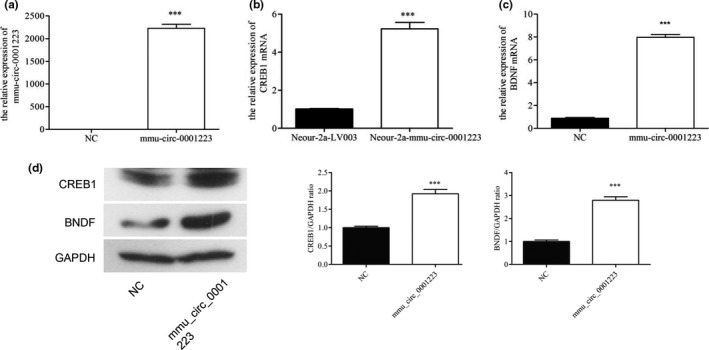
Mmu_circ_0001223 promotes CREB1 and BNDF expression in CUMS mice. (a) Mmu_circ_0001223 expression in PC12 cells transfected with lentivirus vectors. (b, c) CREB1 (b) and BDNF (c) expression in PC 12 cells transfected with lentivirus vectors. Quantitative RT‐PCR was used for determination of gene expression. (d) CREB1 and BDNF protein levels in PC 12 cells transfected with lentivirus vectors. Western blotting was performed and GAPDH was used as the internal standard. NC: negative control; CREB1: cAMP response element binding protein; BDNF: brain‐derived neurotrophic factor; *** refer to a *p* value of <0.001

To clarify the interaction of mmu_circ_0001223 with CREB1 and BNDF expression in the pathophysiological processes of depression as well as depression treatment with SLPN, the mmu_circ_0001223 were over‐expressed in cultured PC12 cells by lentiviral expression system (Figure 6a). Interestingly, we observed that mmu_circ_0001223 overexpression significantly promoted CREB1 expression (Figure 6b). Also, the mRNA level of BDNF in PC12 cells was also greatly increased by the transfection with mmu_circ_0001223‐expressing vectors (Figure 6c). Protein abundance analysis by western blotting further confirmed the regulatory effects of mmu_circ_0001223 on CREB1 and BNDF expression in PC12 cells (Figure 6d). Taking together, these results showed that mmu_circ_0001223‐regualted CREB1 and BNDF expression was involved in the inhibition of depression by SLPN in CUMS mice.

## 4 DISCUSSION

Depression is a complicated mood disorder for which few drugs were available in clinics. To effective screening for novel candidates of anti‐depression drugs, the chronic unpredictable mild stress model was previously established using rats, but later adapted for mice due to the easy availability of transgenic animals, the inter‐strain variability of mice that could be utilized for investigating contribution of genetic background and relatively lower cost (Nollet, Le, & Belzung, 2013). Since then, this mouse model has been successfully applied for the characterization of a number of promising antidepressant candidates, and also led to broader understanding of depression pathogenesis (Goshen et al., 2008; Papp & Moryl, 1994; Xu et al., 2006). As introduced above, natural chemical from traditional herbal medicines have been shown as valuable sources of novel antidepressants, such as curcumin from traditional Chinese medicine Xiaoyao‐san which could reverses the adverse effects of chronic stress on animal behaviors through regulation of hypothalamic‐pituitary‐adrenal (HPA) axis and neurotrophin factor (Xu et al., 2006). Based on our previous revelation of the anti‐depression role of SLPN, the underlying mechanisms were further addressed in the present study using the widely applied chronic unexpected mild stress mice model.

By monitoring mice behaviors via body weight curve, forced swimming and tail suspension tests, SLPN was proved as an effective antidepressant and then used for the following therapeutic efficacy and mechanism investigations. Inspired by the prevalent implications of circular RNAs in various human pathological processes, we comprehensively analyzed the circRNA profiles in CUMS mice with typical depression symptom, and identified a large number of circRNAs that might carry out key roles in depression pathogenesis. In consistence with previous references showing the great changes of circRNA profiles associated with depression (Jiang et al., 2017), we validated here again the drastically altered expression of circRNAs in mice with depression‐like symptoms induced by SLPN. These significant difference of circRNA expression suggested that the circRNA molecules might play key roles in depression pathogenesis. Other large‐scale identifications of circular RNAs in brain tissues and related pathological tissues have also revealed the existence of circRNAs in large quantitation associated with brain development, functions and disorders (Lu & Xu, 2016; Song et al., 2016; Venø et al., 2015). Together with our findings, it could be speculated that circular RNAs might act as key components of regulating networks underlying maintenance of normal neuron functions and dysfunctions.

More interestingly, in the sequencing assay we observed that most differentially expressed circular RNAs in both the mouse ventral medial prefrontal cortex and hippocampus tissues by SLPN were synergistically increased or down‐regulated. This phenomenon indicated that the inhibition of depression by SLPN might be mediated by the coordinated modulation of signaling pathways in these two important organs in brains, which were closely associated with depression and other psychological disorders (Campbell & Macqueen, 2004; Gusnard, Akbudak, Shulman, & Raichle, 2001). The ventral medial prefrontal cortex in the mammalian brain is reported to be responsible for risk and fear processing, emotional responses inhibition and other psychological processes (Boes et al., 2011). The hippocampus in human and other vertebrates is an important part of limbic system that regulates both short‐term and long‐term memory, especially the spatial memory (Buzsáki & Moser, 2013). The synergic functioning and regulation of these two organs in mammalian brains were observed in pleiotropic physiological and pathological events. For instance, previous reference found that the activities of the ventral hippocampus and medial prefrontal cortex were synchronized during anxiety (Adhikari, Topiwala, & Gordon, 2010). The synchronized alteration of major circular RNA expression in these two brain organs induced by SLPN in this study further supported the notion that such synchronization acts as a general mechanism. More importantly, we suggested here that synergistically modulated prefrontal cortex and hippocampus activities could be an important mechanism mediating inhibition of depression by other antidepressants derived from natural bioactive chemicals.

For exemplary demonstration of circular RNAs in inhibition of depression by SLPN, the expression of mmu_circ_0001223 was significantly recovered by SLPN treatment in both the prefrontal cortex and hippocampus tissues of CUMS mice. This also the case with the expression of CREB1 and BNDF, both of which were closely associated with depression (Grønli et al., 2006; Xu et al., 2006). More importantly, the overexpression of mmu_circ_0001223 in PC12 cells by lentiviral system resulted into remarkable increase of CREB1 and BNDF expression, showing that the implication of mmu_circ_0001223 in depression might be mediated by the modulation of CREB1 and BNDF‐related signaling pathways. Moreover, how the expression of CREB1 and BNDF is modulated by this circRNA, how SLPN treatment induced the elevation of mmu_circ_0001223, are remaining scientific topics that deserve further investigation. In view of the large number of differentially expressed circular RNAs identified by our deep sequencing, the studies of other circRNAs would provide novel perspectives on depression pathogenesis, as well as the parts played by circular RNAs. Another enlightenment from our results should be that development of novel drugs targeting circular RNA expression might be effective method of inhibiting depression development in clinics.

In summary, we presented in this study SLPN could significantly inhibit the development of depression in CUMS mice, accompanied with great changes of circular RNA profiles in both the ventral medial prefrontal cortex and hippocampus tissues such as mmu_circ_0001223. Overexpression of mmu_circ_0001223 in PC12 significantly promoted CREB1 and BNDF proteins levels, which might be an important mechanism underlying the inhibition of depression by SLPN. These founding provided valuable insights into depression pathogenesis, as well as the roles of circular RNAs in this process, which could be applied for development of novel antidepressants.

## CONFLICT OF INTEREST

The author reports no conflicts of interest in this work.

## AUTHORS CONTRIBUTIONS

Jun Li conceived and designed the study, and critically revised the manuscript. Hualin Zhang performed the experiments, analyzed the data and drafted the manuscript. Ziming Chen, Zhiyong Zhong and Weifan Gong participated in study design, study implementation and manuscript revision. All authors read and approved the final manuscript.
